# PIEZO2 Proton Affinity and Availability May Also Regulate Mechanical Pain Sensitivity, Drive Central Sensitization and Neurodegeneration

**DOI:** 10.3390/ijms26031246

**Published:** 2025-01-31

**Authors:** Balázs Sonkodi

**Affiliations:** 1 Department of Health Sciences and Sport Medicine, Hungarian University of Sports Science, 1123 Budapest, Hungary; bsonkodi@gmail.com; 2 Department of Sports Medicine, Semmelweis University, 1122 Budapest, Hungary

**Keywords:** hippocampus, Piezo2, Piezo2 channelopathy, proton affinity, ultrafast long-range signaling, vesicular glutamate transporter

## Abstract

The current opinion manuscript posits that not only Piezo2 voltage block, but also proton affinity and availability in relation to Piezo2, a mechanically gated ion channel, may count in the mediation of pain and its sensitivity. Moreover, this paper argues that autonomously acquired Piezo2 channelopathy on somatosensory terminals is likely the initiating peripheral impaired input source that drives the central sensitization of spinal nociceptive neurons on the chronic path as being the autonomous pain generator. In parallel, impaired proprioception and the resultant progressive deficit in neuromuscular junctions of motoneurons might be initiated on the chronic path by the impairment of the proton-based ultrafast proprioceptive feedback to motoneurons due to disconnection through vesicular glutamate transporter 1. The irreversible form of this autonomously acquired Piezo2 ion channel microdamage, in association with genetic predisposition and/or environmental risk factors, is suggested to lead to progressive motoneuron death in addition to loss of pain sensation in amyotrophic lateral sclerosis. Furthermore, the impairment of the proton-based ultrafast long-range oscillatory synchronization to the hippocampus through vesicular glutamate transporter 2 may gain further importance in pain modulation and formation on the chronic path. Overall, this novel, unaccounted Piezo2-initiated protonic extrafast signaling, including both the protonic ultrafast proprioceptive and the rapid nociceptive ones, within the nervous system seems to be essential in order to maintain life. Hence, its microdamage promotes neurodegeneration and accelerates aging, while the complete loss of it is incompatible with life sustainment, as is proposed in amyotrophic lateral sclerosis.

## 1. Introduction

Piezo2 is a transmembrane excitatory mechanosensitive protein with mechanosensitive functions like proprioception, fine touch, tactile pain and interoceptive pressure pulse detection. Nonetheless, our knowledge is still fairly limited when it comes to the precise topology, not to mention all functional aspects of this protein. One not entirely explored but critical aspect of Piezo2 is how exactly external physical cues are translated into mechanistic and functional actions during mechanotransduction. Fortunately, genetically engineered animal studies offer a technological solution for deciphering the mechanistic and biophysical actions of Piezo2.

Sánchez-Carranza et al. recently demonstrated that genetically engineered mutant PIEZO2 mice successfully block the physiological voltage modulation and control of Piezo2 [1]. Earlier, it was shown that mechanical force provides the opening of these channels at high positive voltages; in contrast, they are blocked at resting negative membrane potentials. However, in their study, the alteration of a PIEZO2 residue at an evolutionarily conserved arginine (R2756) site to histidine or lysine increased the sensitivity of nociceptors by diminishing voltage block, but not the mechanical thresholds [1].

The current opinion piece posits that not only PIEZO2 voltage block [1], but also its proton affinity and availability [2] in conjunction might also initiate pain and regulate mechanical pain sensitivity and drive central sensitization and the persistent pain associated with neurodegeneration.

## 2. Piezo2 and Its Schottky Barrier Diode-like Function and Proton Affinity

Piezo2 proteins were first discovered by the Nobel Prize laureate Ardem Patapoutian and his team. Piezo2 and Piezo1 proteins are unique transmembrane mechanotransduction channels with giant and distinctive structures, not to mention the aforementioned functions. The current knowledge of Piezo channels’ physiological role in mechanotransduction is summarized in the excellent review of Bailong Xiao [3], who was also a former member of Ardem Patapoutian’s research team. 

Earlier, it was theorized that Piezo2 is not only a voltage rectifier as part of its semiconductor Schottky barrier diode-like feature with low-frequency noise, but the activation of proprioceptive terminal Piezo2 initiates an unaccounted, novel proton-based ultrafast long-range oscillatory synchronization to hippocampus [4,5]. Hence, this theory emphasizes the link between Piezo2 and protons, where protons act like as neurotransmitters in proprioceptive signaling within the nervous system [4,5]. Indeed, the burst activating and inactivating Piezo2 ion channels carry the enigmatic traits of Schottky barrier diodes with their very fast switching actions. 

It is noteworthy again that the alteration of a PIEZO2 residue at an evolutionarily conserved arginine (R2756) site to histidine or lysine increased the sensitivity of nociceptors by diminishing voltage block [1]. A long-known hallmark of arginine is that it has the highest proton affinity among amino acids [6]. Furthermore, arginine is also lipophilic, hence contributing to cell membrane integrity by lessening its permeability [7]. Therefore, the change of arginine to histidine or lysine not only decreases the proton affinity of PIEZO2, but also diminishes its lipophilicity and increases cell membrane proton permeability at a functionally critical locus. It may carry remarkable importance since the close vicinity of negatively charged lipids surrounding Piezo2 has been proven functionally [4,5]. 

Indeed, Piezo2 carries similar characteristics to Piezo1 when it comes to membrane voltage modulation [8]. However, the intriguing connection of protons and Piezo channels should be considered as well. A supportive finding is that the protonation of PIEZO1 in fact stabilizes the inactivation of this ion channel [9]. An earlier study also showed that swapping arginine in certain structural sites of double mutation eliminates PIEZO1 inactivation [10] and is translated as these mutations resulting in such an energy drop in the inactivated state of the channel that impedes the return to the open state [9]. 

It is important to note that “proton affinity switch” exists in nature as an autonomous light-driven proton pump of bacteriorhodopsin [11]. Even a small change in the proton affinity of bacteriorhodopsin protein results in a non-equilibrium state, and that is sufficient to induce unidirectional transmembrane proton transport despite there being no accessibility path for protons [11]. Accordingly, the swap of amino acids results in replacing arginine with methionine on the extracellular side, while lysine is replaced by arginine on the intracellular side [10]. The current author proposes that this small change in proton affinity induced by the translocation of arginine from the extracellular site of the cell membrane to the intracellular site will result in the aforementioned unidirectional transmembrane proton transport even in the absence of a proton accessibility path. Furthermore, the current author suggests that Piezo2 channels not only resemble Piezo1 in membrane voltage modulation, but in proton affinity characteristics as well. In support, a recent paper posits that oxaliplatin, a third-generation platinum derivative chemotherapeutic agent, may also induce the aforementioned small change in proton affinity or a “proton affinity switch” on Piezo2, leading to Piezo2 channelopathy, due to the enigmatic high-proton-affinity nature of platinum [2]. It is important to note that this paper also proposes that the aforementioned energy drop is the direct result of the “proton affinity switch” due to the intracellular metabolic shift from high-energy-generating glycolysis pathways to evolutionarily conserved lower-energy-generating glutamine fermentation pathways [2]. 

Moreover, Piezo2 is not viewed as a ligand-gated ion channel; however, its auxiliary proteins, like myoblast determination protein 1 (MyoD) [12] and transmembrane protein 120A (TMEM120A)/TACAN [13], certainly have functional roles. It has been proposed that these auxiliary proteins go through conformational changes under allostatic stress that lead to their dissociation from Piezo2 and to the resultant acquired Piezo2 channelopathy [4]. This conformational dissociation from Piezo2 may also provide the aforementioned unidirectional transmembrane proton pathway in an acquired fashion when no proton flux should exist from the extracellular membrane surface into intracellular territories.

It is noteworthy that a semiconductor Schottky barrier diode is formulated by the junction of a semiconductor with a metal. Part of the aforementioned theory is that the extracellular matrix functions like a semiconductor modulator, with the active contribution of, e.g., syndecans as proton collectors and distributors [4]. In addition, transitional mitochondrial transportation and pattern changes provide not only the neural operational energy, but also the availability of high-frequency oscillations within the patterned mitochondrial networks propelled by Huygens synchronization [5] and the metal ion density for the Schottky barrier diode-like function. 

It has been also hypothesized that Piezo2 channelopathy could be autonomously acquired, not only inherited or genetically engineered, in an acute transient form in the primary damage phase of delayed-onset muscle soreness (DOMS) by fatiguing forced lengthening contractions under allostatic stress [14]. Accordingly, this bi-phasic and bi-compartmental DOMS mechanism is proposed to be initiated by the primary damage of intrafusal proprioceptive terminal Piezo2 but is suggested to involve all four available somatosensory fiber types in muscles, reflected in an acute axonopathy [14], namely large Type Ia proprioceptive fibers with microdamaged Piezo2 content and Type II fibers in the intrafusal compartment and small Type III and nociceptive C-fibers in the extrafusal muscle compartment where Piezo2 content is also suggested to go through acquired channelopathy in the secondary damage phase of DOMS [14]. Correspondingly, the primary damage of the intrafusal proprioceptive Piezo2 is like an explosion of an airbag and the resultant loss of airbag protection. As a result, Piezo2 channelopathy decreases the proprioceptive protection of the extrafusal compartment. This leads to peripheral tissue damage, including extracellular matrix damage, that in turn impairs Piezo2 mechanogating on Type III fibers [14]. This is equivalent to the lost semiconductor modulating capability in the aforementioned semiconductor Schottky barrier diode-like feature of Piezo2. 

## 3. The Role of Piezo2 in Sensitization and Pain

However, not only this bi-compartmental crosstalk of Piezo2-containing somatosensory neurons is intriguing in DOMS. Mechanical sensitization is also an important aspect of this mechanism through the proposed activation of wide-dynamic-range (WDR) neurons on the spinal dorsal horn by imbalanced subthreshold Ca^2+^ currents, N-methyl-d-aspartate (NMDA) activation and L-type Ca^2+^ currents [4]. It is notable that the progressive degenerative irreversible type of acquired Piezo2 channelopathy at proprioceptive terminals is suggested to be the gateway to pathophysiology in amyotrophic lateral sclerosis (ALS) where this proposed WDR activation mechanism is abrogated due to the theorized irreversibly lost Piezo2 function [4]. This irreversible Piezo2-channelopathy-derived lost WDR activation is proposed as the underlying reason why ALS used to be considered as a painless neurodegenerative lethal disease [4]. In support, loss-of-function mutations in PIEZO2 result in a loss of pain and sensitization [15].

In light of the finding of Sánchez-Carranza et al. [1], it seems that decreased proton affinity of an altered PIEZO2 residue instigates earlier voltage block and increased sensitivity. Moreover, oxaliplatin is suggested to have an analogous effect [2]. In addition, the diminished lipophilicity and resultant increased cell membrane permeability, or the theorized ‘leakiness’ [14], induce undesired unidirectional proton flux from the cell surface into intracellular space that imbalances subthreshold intracellular Ca^2+^ currents. These imbalanced subthreshold intracellular Ca^2+^ currents contribute to WDR neuron activation on the spinal dorsal horn [4]. In support, intracellular Ca^2+^ currents are significantly more sensitive to intracellular protons than extracellular ones [16]. 

An additional consideration of the ultrafast proprioceptive signaling and the rapid pain system of the nervous system is the proposed proton tunneling, as one form of quantum tunneling, through vesicular glutamate transporter 2 (VGLUT2) [5]. Accordingly, the large fiber intrafusal primary afferents are suggested to be the low-frequency oscillatory ultrafast proton signalers, while the small fibers are the rapid ones. Moreover, these large fiber primary afferents may not only provide proton-based low-frequency coupled synchronization to the hippocampus, but also provide Piezo2-modulated proton-based ultrafast fine grading of proprioception to motoneurons [5]. As a tangential remark, the impairment of this peripheral Piezo2-initiated muscle spindle–hippocampal pathway will gain further importance when pain, or Piezo2 channelopathy, takes a chronic path. In support, it has long been known that functional abnormalities of the hippocampus are evident in the presence of chronic pain [17].

## 4. The Role of Piezo2 in Proprioception and Its Impairment

Ardem Pataputian and his team showed that Piezo2 is the principal mechanosensitive ion channel responsible for proprioception [18], but it is important to note that it is not the only one [19]. Correspondingly, the current author proposes that this principality in regard to proprioception stems from Piezo2’s emblematic capability of initiating the aforementioned protonic ultrafast signaling to the hippocampus. Proprioceptive neurotransmission is not lost even in the absence of this Piezo2-initiated ultrafast signaling, but it is taken over, for example, by Na_v_1.1 ion channels in a miswired fashion [2].

**Figure 1:**
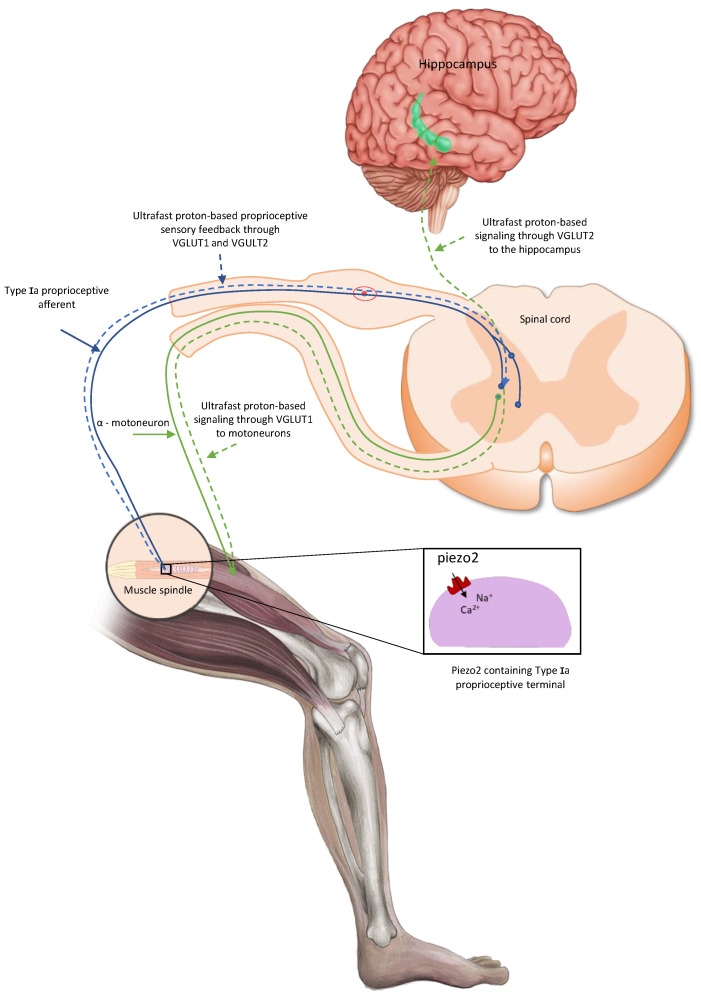
PIEZO2-driven, proton-based ultrafast signaling from intrafusal proprioceptive terminals, and how it links motoneuron activity via VGLUT1 and hippocampal theta rhythm via VGLUT2 [21].

Indeed, Piezo2 is present on Type Ia large fiber proprioceptive terminals [19]. The Piezo2-containing proprioceptive large fibers are also suggested to provide proton-tunneled ultrafast mechanosensory feedback to motoneurons through vesicular glutamate transporter 1 (VGLUT1) (Figure 1) [4], and that is theorized to be transiently impaired in DOMS and come to an end in ALS [4]. This transient disconnection of proprioceptors on motoneurons through VGLUT1 is proposed to be reflected in the significant delay of the medium-latency response of the stretch reflex in DOMS [4,5]. As a background, the monosynaptic static phase firing sensory encoding is suggested to be impaired due to Piezo2 channelopathy [20]. Hence, Piezo2-initiated proton-based signaling through VGLUT1 likely provides the monosynaptic static phase firing sensory encoding of the stretch reflex (Figure 1) [4,5]. 

The proposed acquired Piezo2-channelopathy-induced impaired Type Ia monosynaptic excitatory postsynaptic potential also activates NMDA receptors [14], hence further contributing to WDR neuron activation [4]. In addition, it is suggested that Piezo2 provides proton handling to acid-sensing ion channels 2 (ASIC2) intrafusally and likely to acid-sensing ion channels 3 (ASIC3) extrafusally, and in return, ASIC provides proton handling for VGLUT proton tunneling [5]. The impairment of proprioceptor Piezo2-initiated proton handling to ASIC2 is translated to be the cause of ASIC2 upregulation in motoneurons in ALS [4]. It should be pointed out that static phase firing sensory encoding of the stretch reflex is not lost due to the DOMS effect, but signaled through Type II proprioceptive fibers, probably through ASIC3, in a polysynaptic way instead of a monosynaptic way [14]. However, keeping the static phase firing sensory encoding on only polysynaptic transmission, the equivalent of lost proton-based ultrafast long-range signaling, wears out the neuromuscular junction of motoneurons, leading to motoneuron degeneration or accelerated aging, and even causes motoneuron death, like in the case of ALS [4]. 

## 5. The Role of Piezo2 in Neurodegeneration

The aforementioned relevance of negatively charged lipids in cellular membranes and on membrane surfaces surrounding the close vicinity of Piezo2 has been highlighted functionally [4,5]. So far, the most compelling research finding on how Piezo2 has a role in neurodegeneration is reflected in Angelman syndrome (AS). AS is a neurogenetic disorder with associated neurodegeneration, and the symptoms are as follows: delayed development, intellectual disability, impaired coordination, poor balance and gait ataxia [22]. It is important to emphasize that the neurogenetic aspect of this disease does not directly relate to PIEZO2. The replacement of linoleic acid improves the mechano-excitability of Piezo2, leading to improved gait function of AS mice [22]. Romero et al. proposed that the lost expression of UBE3A, an E3 ubiquitin-protein ligase, causes AS in association with Piezo2 dysfunction [22] or acquired Piezo2 channelopathy. Moreover, they successfully found that a particular fatty acid supplementation, namely linoleic acid, improved Piezo2 function and mitigated the mechanosensory deficits of AS [22].

An intriguing question is how linoleic acid has the above positive impact on the proposed Piezo2 channelopathy. Linoleic acid is probably not a mediator of TMEM120A/TACAN and hence has its unknown finetuning action directly on Piezo2 [23]. Since TMEM120A/TACAN is capable of regulating gene expression [24], like MyoD is a myogenic transcription factor involved in early regeneration [25], and Piezo2 channelopathy is proposed to be a principal transcription activator [4], the current author suggests that linoleic acid stabilizes the functional protein–lipid interaction of Piezo2. This effect is probably due to the highly lipophilic nature of linoleic acid. Correspondingly, linoleic acid likely prevents the occurrence of the aforementioned unidirectional proton ‘leak’ pathway that is suggested to be induced by the dissociation of TMEM120A and/or MyoD from Piezo2 as a consequence of acquired Piezo2 channelopathy. After all, this impediment of the unidirectional proton ‘leak’ by linoleic acid explains the restitution of Piezo2 function and the elimination of the neurodegenerative aspect of acquired Piezo2 channelopathy in AS. However, it is important to note that this potential therapeutic theory should be validated in future studies. It is noteworthy that MyoD gene transfer lessens survival and facilitates motor neuron degeneration and muscle denervation in the G93A SOD1 fALS mouse model [25], most likely due to the inhibitory function of MyoD on still-available functional Piezo2 and the facilitation of the already depleted regenerative capacity in ALS.

Moreover, Lalancette-Herbert et al. demonstrated that Type Ia proprioceptive sensory afferents from the muscle spindle contribute to alpha motor neuron degeneration [26]. In support, an ALS theory proposed that the proprioceptive terminal microdamage-induced ‘switch’ of static phase firing encoding of Type Ia fibers to Type II fibers results in a delay of the medium-latency response (MLR) of the stretch reflex, and later, this delay of MLR was verified in a DOMS study, and it was theorized that Type Ia monosynaptic input is lost due to the aforementioned proton-based signaling disconnection through VGLUT1 on motor neurons [4]. Accordingly, McIntosh et al. demonstrated in an ALS mouse model that dysfunctional abnormalities happen first on neuromuscular junctions, followed by postsynaptic structural detachment from the neuromuscular junctions, but all of this happens before motor symptoms develop in ALS [27]. 

The current author attributes these dysfunctional abnormalities and detachment to microdamaged Type Ia terminal-derived lost static phase firing encoding via lost proton-based signaling through VGLUT1 on alpha motor neurons. Consequently, the current author suggests that Type Ia excitatory input contributes to alpha motor neuron degeneration, as suggested by Lalancette-Herbert et al. [26], by glutamate excitotoxicity due to progressive irreversible Piezo2-channelopathy-associated lost vesicular glutamate release at Type Ia terminals [4]. This resultant glutamate excitotoxicity induces persistent inward currents on alpha motor neurons due to the proton-based signaling disconnection through VGLUT1 [4], eventually leading to the degeneration of alpha motor neurons [26]. It is remarkable that an analogous impairment of Type Ia proprioceptive afferent input has been detected in the early stage of another neurodegenerative disease, namely in spinal muscular atrophy (SMA) [28,29]. 

## 6. Concluding Remarks

The published finding of Sánchez-Carranza et al. [1] is a major step forward, showing that somatosensory acquired Piezo2 channelopathy is likely the initiating peripheral impaired input source that drives the central sensitization of spinal nociceptive neurons on the chronic path [14,30,31]. Hence, central sensitization independent of peripheral input does not seem to be the autonomous pain generator [30,31], but rather the autonomously acquired Piezo2 channelopathy. Over and above, it seems that dysfunctional Piezo2-induced impaired proton signaling contributes to mechanical sensitization and pain, even more so in the mechanical hyperalgesia of DOMS in a bi-phasic and bi-compartmental manner, but it appears that the progressive degenerative complete loss of this likely principal and vital Piezo2-initiated proton-based neurotransmission is also the proposed gateway to ALS pathophysiology [4]. The scientific community is aware that acute intensive exercise events often cannot be translated by classic neurotransmitters time-wise; therefore, this Piezo2-initiated ultrafast proton-based signaling within the nervous system may fill this gap, and research exploring this subject is warranted. 

This theorized Piezo2-initiated proton-based signaling has relevance in ontogeny, as when human ancestors developed bipedalism, postural control demanded a higher level of Piezo2-supported proprioception. Correspondingly, mechanical pain, as an alarm signal feedback, also carries evolutionary importance, not to mention that DOMS allows 6–8 h of an initial relative pain-free period right after the proposed primary damage in order to escape from danger under largely unimpaired proprioception and the absence of pain limitation. It is promising that low-frequency noise of Schottky barrier diodes should be detected, and accordingly, it is suggested that the low-frequency power of heart rate variability reflects the activity level of Piezo2 in the baroreceptors [5]. On a basic level, autonomously impaired Piezo2-function-induced mechanical sensitization and pain likely alarm us that the conversion of external physical cues into inner biological ones through mechanotransduction is malfunctional, while the irreversible loss of it is incompatible with life, as proposed to be the case in ALS [4]. 

In summary, this novel, unaccounted Piezo2-initiated protonic extrafast signaling, including both the ultrafast and rapid forms of signaling, within the nervous system seems to be essential in order to maintain life; hence, the impairment of it leads to neurodegeneration or accelerates aging, whereas the complete loss of this channel activity is incompatible with life sustainment.
